# Living a bacterial lifestyle as an academic researcher

**DOI:** 10.1093/femsml/uqac012

**Published:** 2022-08-01

**Authors:** Sarah Wettstadt

**Affiliations:** GmbH MicroComms, Berlin, Germany

## Abstract

About the scientific life of Melanie Blokesch

## Acquiring superpowers

Melanie Blokesch has always been extremely motivated by acquiring new knowledge and skills. Her original idea was ‘to study biology with the goal to potentially end up in cancer research’. However, she was instantly amazed by microbes and their abilities to such an extent that she instead chose microbiology and genetics during her time at University. It even got her to do a PhD at the Ludwig-Maximilians-University in Munich, Germany. Here, she investigated how *Escherichia coli* uses hydrogenases with unique Nickel-iron-active centres to produce hydrogen (Böck et al. 2006). In her first first-author paper, she characterised a small accessory protein that participates in the maturation process of [NiFe]-hydrogenases in *E. coli* and described their novel biochemical function (Blokesch and Böck 2002).

After her PhD, Melanie moved to the Department of Microbiology & Immunology at Stanford University, California, and switched to the pathogen *Vibrio cholerae*. This causative agent has been responsible for several cholera pandemics worldwide with the 7th pandemic ongoing since 1961. Being naturally competent, the species *V. cholerae* likes to acquire genetic material and thus new skills from its surrounding. In her research at Stanford, Melanie focused on competence regulation (Meibom et al. 2005) and how transformation can trigger serogroup conversion (Blokesch and Schoolnik 2007) of *V. cholerae*. As Melanie kept exploring how *V. cholerae* gains new superpowers with its competence machinery, she advanced her own capabilities to that of an Assistant Professor at the Global Health Institute at the Swiss Federal Institute of Technology in Lausanne (EPFL), Switzerland.

## Establishing a niche

In Lausanne, Melanie became interested in the DNA uptake machinery of this pathogen (Seitz and Blokesch 2013). In a study that she considers ‘one of the most complete and elegant work from [my] her lab’, her team unravelled how a periplasmic protein translocates foreign DNA across the outer membrane (Seitz et al. 2014). They also started investigating how *V. cholerae* adheres to and settles down on biotic surfaces in their natural aquatic environments. With that new research focus, she and her team found a link between the pathogen's killing weapon and its competence machinery. It was already known that those *V. cholerae* strains that are not responsible for the currently ongoing 7th pandemic of cholera use their antibacterial Type 6 Secretion System to fire lethal toxins into neighbouring competitors. And if these competitors—either bacteria or amoeba—were lacking the corresponding immunity proteins to the fired toxin, they are unlikely to survive the attack. To the surprise of the microbiology community, pandemic *V. cholerae* strains co-regulate their Type 6 Secretion System with the competence regulon when growing on chitinous surfaces. In this major publication, Melanie and her team suggested that *V. cholerae* would first get rid of microbial competitors by killing them and then take up the dead microbe's DNA (Borgeaud et al. 2015).

But *V. cholerae* is not able to kill them all. Interestingly, some bacteria living in the intestinal tracts of animals are resistant to *V. cholerae*’s Type 6 Secretion System attacks. These bacteria might contribute to the colonization barrier that usually protects the host from the devastating cholera disease. Melanie and her team explored the underlying mechanisms of this resistance strategy. They found that members of the *Enterobacter cloacae* complex and the *Klebsiella* genus achieved resistance against *Vibrio*’s Type 6 Secretion System attacks without the help of immunity proteins. Indeed, these human gut commensals rely on superior molecular weapons or their capsular polysaccharides as physical barriers around their cells—a new defensive mechanism within microbial communities (Flaugnatti et al. 2021).

Learning more and more about how *V. cholerae* settles down in its environment, this very same research topic helped Melanie establish her own niche within the *Vibrio* community. As an independent researcher, always having her previous projects in mind, Melanie then ‘extended the research topics based on interesting experimental observations and open questions that [she] identified in the literature’. Hence, with more incoming grants, she could tackle a larger array of topics and spread out to new directions over the years. Eventually, this success led to her election to prestigious societies like the European Academy of Microbiology (EAM), European Molecular Biology Organisation (EMBO), the German National Academy of Sciences Leopoldina and the American Academy of Microbiology (AAM).

## Interacting with the environment

Yet, being a full professor didn't mean for Melanie to leave the lab bench, since she enjoys the immediate and quick results from experiments. Indeed, she still ‘checks plates in the incubator in the morning before even having [her] first coffee’ and works closely with her technician who's performing experiments under her guidance. Melanie even says that ‘what seemed annoying as a student or postdoc, such as pouring agar plates or re-streaking hundreds of colonies, becomes almost meditation as a PI’. One can also follow that passion on her Twitter account, where every now and then she publishes photos or anecdotes from her recent lab experiences.

That passion and persistence also led Melanie to her most recent large project. She finally decided to tackle a problem that has been keeping her puzzled for many years. Ever since she started working with *V. cholerae*, she realised that while this bacterium has no issues taking up free foreign DNA, it ‘doesn't do so well with plasmids’. However, her mindset taught her—and now she teaches her lab members the same: ‘don't just look for the expected results and be disappointed when things turn out in a different way; because that's often when the more interesting findings emerge that you should definitively follow up on’. Even though she usually told new lab members to avoid plasmids and add everything onto the chromosome, it was always in the back of her mind that something must be going on. During her postdoc, she already ‘saw some weird morphological changes in plasmid-carrying *V. cholerae* when imaged during exponential phase’.

Then, after a general discussion with a friend and colleague on Vibrios, she had two lab members ‘taking on the challenge to actually address the question of why plasmids are badly tolerated’ by *V. cholerae*. After testing many different strains, they showed that it is ‘exclusively the 7th pandemic clade of *V. cholerae* that doesn't tolerate plasmids’ and they managed to unravel the molecular mechanisms behind it. This study was recently published (Jakólska et al. 2022) and shows once again that the unexpected often results in the most exciting discoveries. Having studied *V. cholerae* and its abilities to acquiring genetic material and establishing a niche, Melanie has finally tools to engineer new strains to study them. Hopefully, this will bring us closer to understanding this devastating bacterium and the disease it causes!
