# Correction to: Living a bacterial lifestyle as an academic researcher

**DOI:** 10.1093/femsml/uqac017

**Published:** 2022-10-12

This is a correction to: Sarah Wettstadt, Living a bacterial lifestyle as an academic researcher, microLife, Volume 3, 2022, uqac012, https://doi.org/10.1093/femsml/uqac012

The originally published version of this manuscript contained an incorrect reference to *Seitz et al. 2014*.

The correct reference is as follows:

Jakólska M, Adams DW, Blokesch M. Two defence systems eliminate plasmids from seventh pandemic Vibrio cholerae. Nature 2022; 604:323–329.

This error has been corrected.

